# Autophagy flux inhibition, G2/M cell cycle arrest and apoptosis induction by ubenimex in glioma cell lines

**DOI:** 10.18632/oncotarget.22594

**Published:** 2017-11-21

**Authors:** Liping Han, Yongfei Zhang, Shuai Liu, Qingwei Zhao, Xianhong Liang, Zhiguo Ma, Prakash K. Gupta, Miaoqing Zhao, Aihua Wang

**Affiliations:** ^1^ Department of Neurology, Qianfoshan Hospital Affiliated to Shandong University, Jinan, P.R. China; ^2^ Department of Neurology, Shandong Police Hospital, Jinan, P.R. China; ^3^ Department of Dermatology, Qianfoshan Hospital Affiliated to Shandong University, Jinan, P.R. China; ^4^ Department of Urology, Shandong Provincial Hospital Affiliated to Shandong University, Jinan, P.R. China; ^5^ Certifying Scientist, Lab Solutions LLC, Atlanta, GA, USA; ^6^ Department of Pathology, Shandong Provincial Hospital Affiliated to Shandong University, Jinan, P.R. China

**Keywords:** glioma, ubenimex, G2/M phase arrest, autophagic flux, apoptosis

## Abstract

This study aimed to investigate whether ubenimex could work as an anti-tumor drug alone in glioma cells and figure out the underlying potential mechanisms. Ubenimex is widely used as an adjunct therapy in multiple solid cancers. However, it is rarely used to treat glioblastoma. The function of ubenimex in enhancing JQ1 treatment sensitivity of glioma cells by blocking autophagic degradation of HEXIM1 was previously studied. However, the detailed mechanism of autophagy regulation by ubenimex remains unclear. The U87 and U251 cell lines were treated with different doses of ubenimex. Cell viability was measured by using the WST-8 assay. Cell death was assessed using trypan blue staining and flow cytometry. The migration and invasive ability of glioma cells were examined by transwell migration/invasion assay. LC3-GFP-RFP was used to measure autophagic flux. Protein expression was assessed by Western blot analysis. Autophagosomes were evaluated using the transmission electron microscopy. Moreover, cell cycle arrest (PI Staining) was measured by flow cytometry. Results revealed that ubenimex inhibited cell proliferation as well as migration/invasion in glioma cells. Besides, ubenimex increased glioma cell death via autophagic flux inhibition. Meanwhile, ubenimex induced G2/M phase arrest and apoptosis, and this effect was accompanied by the decreased levels of p-Akt, indicating the role of ubenimex in the regulation of glioma cell proliferation and metastasis. To sum up, this study concluded that ubenimex could work as an anti-tumor drug alone in the glioma cells via inhibiting autophagic flux and inducing G2/M arrest as well as apoptosis.

## INTRODUCTION

Glioma is the most common neuroectodermal brain tumor, accounting for 50%–55% of the total brain tumors [1]. Despite the development of different treatment strategies, the median overall survival of patients with the most malignant form of glioma still remains about just 12–15 months [2, 3]. Moreover, removing glioma through usual surgery is very difficult because of the extensive formation of microsatellites in the normal brain tissue [4]. Therefore, development of therapeutic interventions for glioma is in greater need than ever before.

It has been shown that Aminopeptidase N (APN, cell surface molecule CD13) is involved in various cellular processes but not limited to cell cycle control, cell motility, cell differentiation, angiogenesis, cellular attachment, and invasion/metastasis of various cancers [5]. Ubenimex has been shown to be a potent APN inhibitor therefore making it widely used as an adjunct therapy after surgery or target therapy [6, 7]. However, there are very few reports that discussed the efficacy of ubenimex in glioma.

Autophagy has been shown to play a vital role in degrading the damaged organelles and providing energy for survival of the cancer cells under treatment. Inhibition of autophagy enhances apoptosis induced by proteasome inhibitor in human glioblastoma U87and U251 cells [8]. Our previous study demonstrated that ubenimex could enhance glioma's JQ1 sensitivity by blocking autophagic degradation of HEXIM1, indicating the importance of autophagy in glioma treatment [9]. However, the detailed mechanism of autophagy regulation by ubenimex remains unclear. Autophagic flux is crucial for the assessment of dynamic autophagy process in a variety of systems and is defined as a measure of autophagic degradation activity. The assays that monitor autophagic flux, or degradative completion of autophagy remains vital to this process. Studying autophagy flux helps to distinguish whether the increase in autophagosomes was due to autophagic activity induction or from the increase in autophagosomes caused by reduction in lysosomal turnover. From that we can further demonstrate the detail role of autophagy inhibition by ubenimex. It has been shown that inhibition of autophagic flux lead to anti-cancer effect in hepatocellular carcinoma cells [10]. However, whether ubenimex could inhibit autophagic flux in glioma needs to be verified.

Via induction of G2/M cell cycle arrest, ubenimex has been shown to have anticancer effects in hepatocellular carcinoma [11]. This effect also needs to be verified in gliomas. Recently, whether cell cycle is associated with cross talk in autophagy regulation has become the hottest topic. Throughout cell cycle, levels of autophagic flux were different among different the phase. Autophagy is active in early mitosis (G1) as well as S phase compared to G2/M phases, this might contribute to helping damaged organelles degradation as well as providing extra energy for surviving under treatment [12]. As cancer cells are arrested in G2/M phase by ubenimex, the role of ubenimex in autophagic flux regulation should be elucidated, also, the relationship between autophagy flux and cell cycle arrest need to be studied.

Akt plays an important role as a classical signaling pathway in the regulation of tumor cell death [13]. When referred to cell death, we need to remember the auophagy-related cell death and apoptosis. Also, the Akt signaling pathway plays a vital role in the connecting apoptosis with autophagy [14, 15]. Our previous study confirmed that ubenimex induces cell death in renal cell carcinoma (RCC) and prostate cancer cells, which was related to the APN activity inhibition and autophagic cell death [16, 17]. PI3K/Akt/mTOR signalling pathway regulates apoptosis and autophagy via a complicated crosstalk. Activation of this pathway allows cellular inhibition of autophagy, resulting in apoptosis. We have previously demonstrated that via Akt pathway inhibition, protective autophagy caused sorafineb resistance in RCC, resulting in autophagic cell death as well as apoptosis. This in turn contributes to the sensitivity of this target treatment [18].

Therefore, this study aimed to investigate whether ubenimex could still work as an anti-cancer drug in glioma and figure out the underlying potential mechanisms.

## RESULTS

### Ubenimex inhibited cell proliferation and induced cell death in U87 and U251 cells

**Figure 1:**
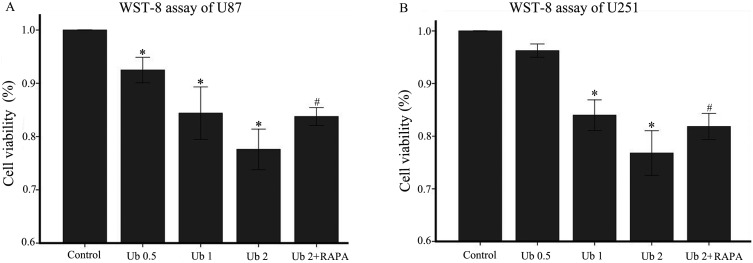
Ubenimex inhibited proliferation and cell death of U87 and U251 cells (**A** and **B**) WST-8 proliferation assay was performed after a 24-h culture of U87 or U251 cells with the indicated concentrations of ubenimex. ^*^P < 0.05, for untreated control cells compared with all ubenimex doses. ^#^*P* < 0.05, for cells treated with 2 mg/mL ubenimex+ rapamycin compared with those treated with ubenimex alone (2 mg/mL). Data are expressed as means ± standard deviation of three independent experiments.

**Figure 2:**
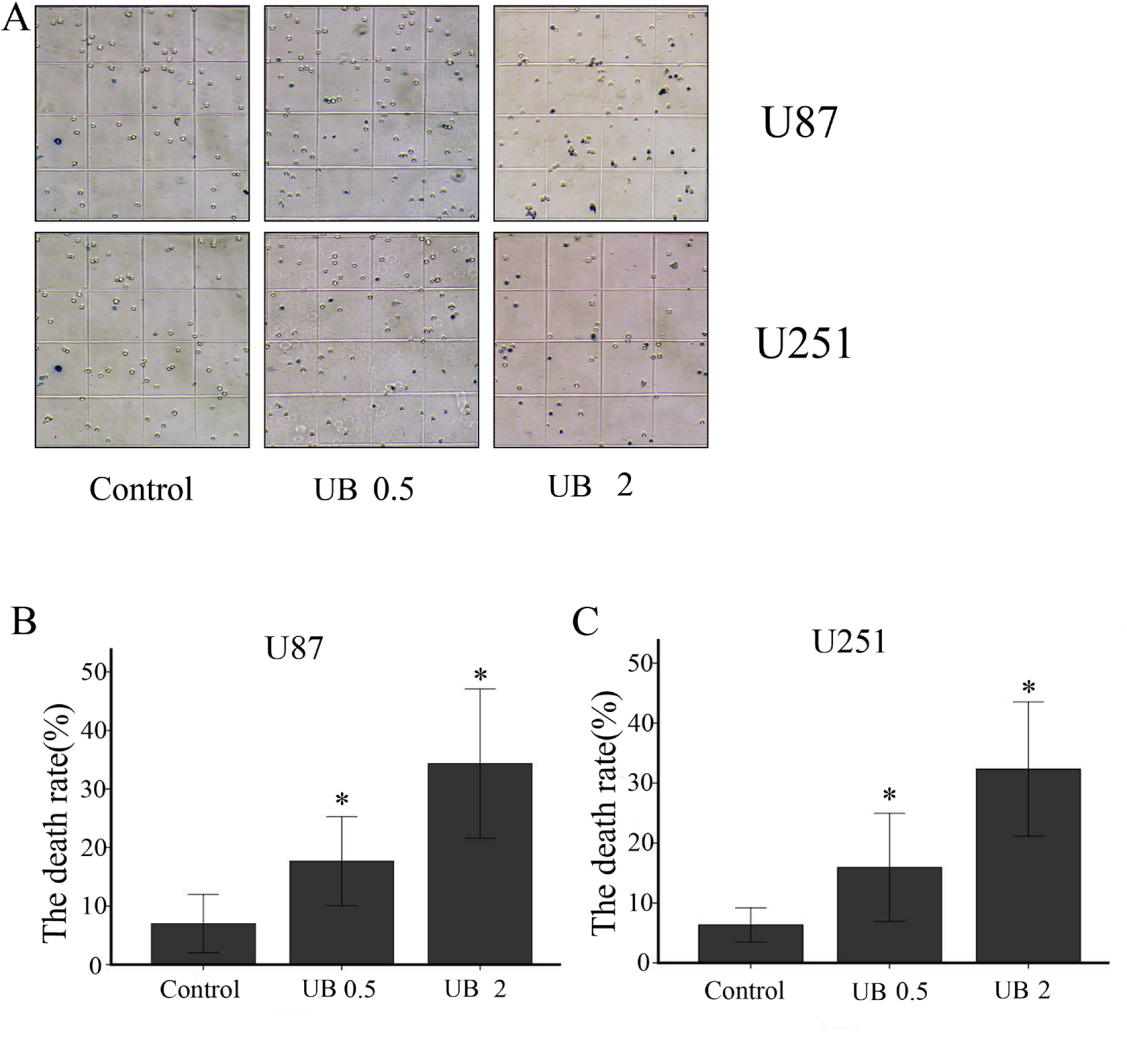
(**A**) U87 and U251 cells were treated with ubenimex at doses of 0.5 and 2 mg/mL for 16 h. Microscopy was used to examine cell death. The death rates of (**B**) U87 and (**C**) U251 cells per field of a high-power microscope. ^*^P < 0.05 versus the control. Data are expressed as mean ± standard deviation of three independent experiments.

WST-8 was used to examine cell proliferation after treatment with different doses of ubenimex in glioma cells (Figure 1). Proliferation of U87 and U251 cells were significantly inhibited by different doses of ubenimex in an increasing dose-dependent manner after 16 or more hours of exposure (*P* = 0.021 and similar response was observed in dose dependent manner. Trypan blue staining was used to count the cell death after treatment with different doses of ubenimex (Figure 2). Similar results were obtained as above. The rate of cell death was increased with the increased dose of ubenimex. So, these results suggested that ubenimex effectively inhibited cell proliferation and induced cell death in both U87 and U251 cells.

### Ubenimex inhibited migration and invasion in glioma cells

**Figure 3:**
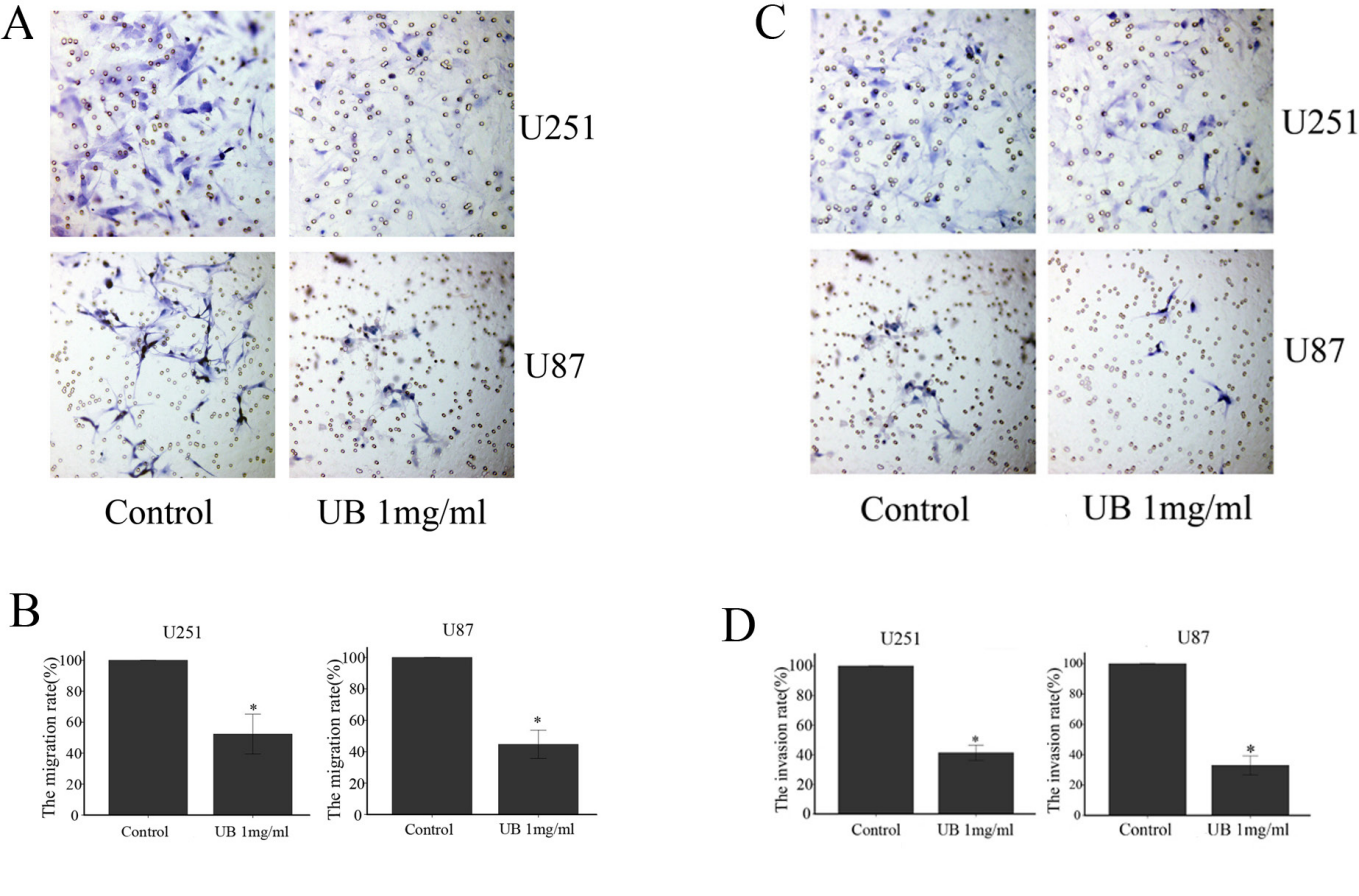
Ubenimex reduced the U87 AND U251 migration and invasion ability Transwell migration and invasion assays were performed to determine the migration and invasion of glioma cells. (**A** and **C**) The images above are shown after the indicated times of culture in control medium or different doses of ubenimex as described above. (**B** and **D**) Quantifcation was performed by counting the percentage of cells that passed through the small well vs control using a high-power microscope. These values are expressed as means ± SD of 3 independent experiments. ^*^P < 0.05 vs. the untreated control cells.

Transwell assays were performed to determine whether ubenimex affected the migratory and invasive capacity of U87 and U251 cells (Figure 3). Migratory capacity of both the cell lines were significantly attenuated by ubenimex in a concentration-dependent manner after 16 or more hours of exposure (*P* = 0.012). Moreover, glioma cells are affected by the dose of ubenimex which plays an important factor therefore, further analysis was performed to see the effect of ubenimex on the invasive activity of both cells using Matrigel invasion assays. Invasive capacity of both the cells were markedly inhibited by pretreatment of ubenimex (*P* = 0.011). Taken together, these results suggest that ubenimex inhibited the migration and invasion of glioma cells.

### Ubenimex worked as an APN inhibitor in glioma cells

**Figure 4:**
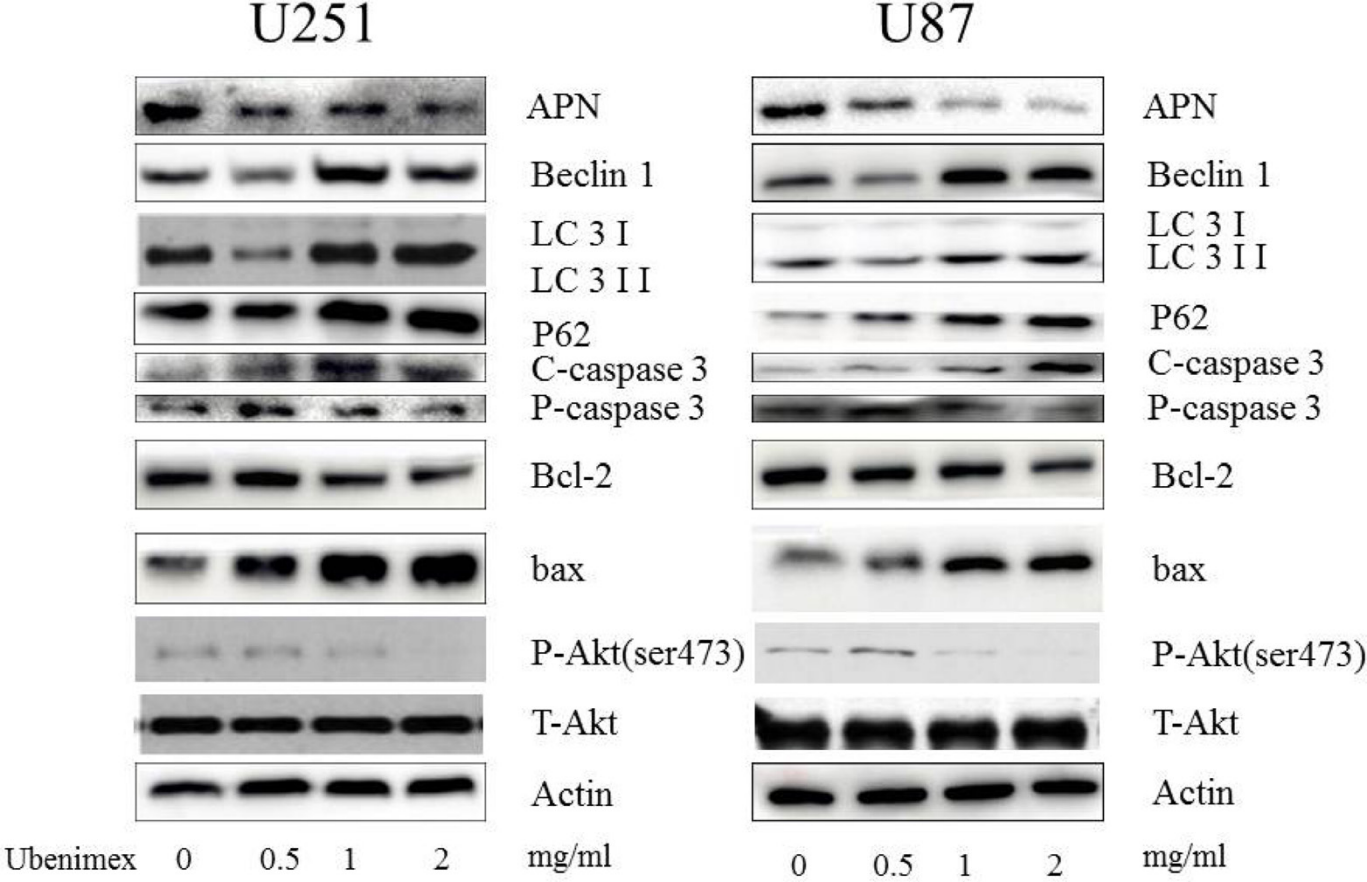
Western blot showed that high dose (1, 2 mg/ml) of ubenimex induced LC-3II/I expression, while low dose (0.5 mg/ml) inhibited it Same trend in Beclin1 expression. These results suggested well connection of ubenimex dose with autophagy, the expression of P62 was gradually decreased with increased dose of ubenimex. Akt pathways and APN were influenced by ubenimex. p-Akt-ser473 was down regulated in a dose-dependent manner. But the total Akt expression showed no difference. Ubenimex, an APN inhibitor, also had an effect on U87 and U251 cells, with a reverse trend for cleaved caspase-3 levels and significantly inhibited the rate of bcl-2/bax.

Western blot analyses were used to test the APN (CD13) expression in the glioma cells following treatment with ubenimex (Figure 4). After 16 h treatment with ubenimex, the APN expression was significantly attenuated in a dose-dependent manner in the glioma cells. Thus, ubenimex functioned as an APN inhibitor in glioma cells.

### Through autophagic flux inhibition, ubenimex influenced the level of autophagy in a dose-dependent manner

**Figure 5:**
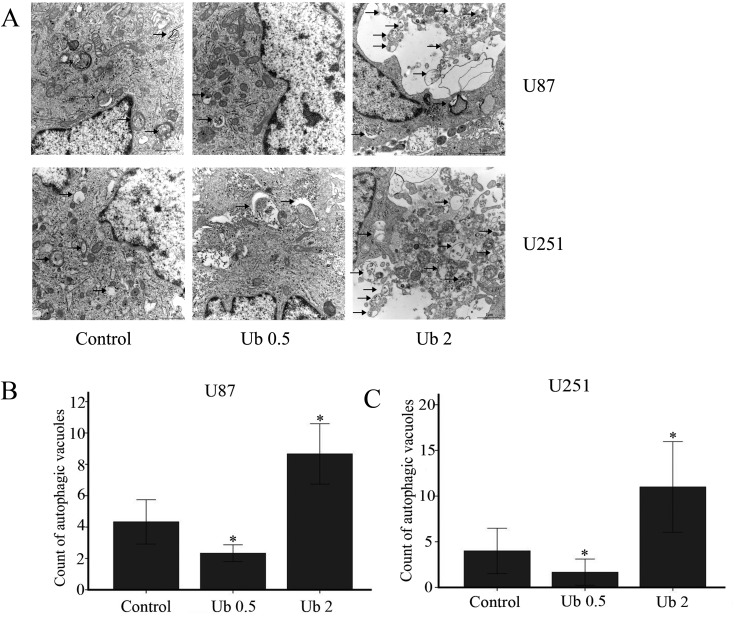
Ubenimex influenced the level of autophagy in U87 and U251 cells (**A**) U87 and U251 cells were treated with ubenimex at doses of 0.5 and 2 mg/mL for 16 h. Electron microscopy was used to examine the number of autophagic vacuoles. (**B**, **C**) The number of autophagic vacuoles in the U87 and U251 per cell (average of five cells) was determined. ^*^P < 0.05 versus the control. Data are expressed as the mean ± standard deviation of three independent experiments.

To examine the level of LC-3 expression in glioma cells following treatment with ubenimex, Western blot and electron microscopy were performed (Figures 4 and 5). A high dose (1, 2 mg/ml) of ubenimex induced LC-3II/I expression, while low dose (0.5 mg/ml) inhibited it. Same trend in Beclin1 expression. These results suggested well connection of ubenimex dose with autophay.

But when western blot was used to analyze the expression of P62, a marker of autophagolysosomal level, the expression was gradually decreased with increased dose of ubenimex (Figure 4). This suggested that ubenimex decreased P62 degradation and the level of autophagy.

**Figure 6:**
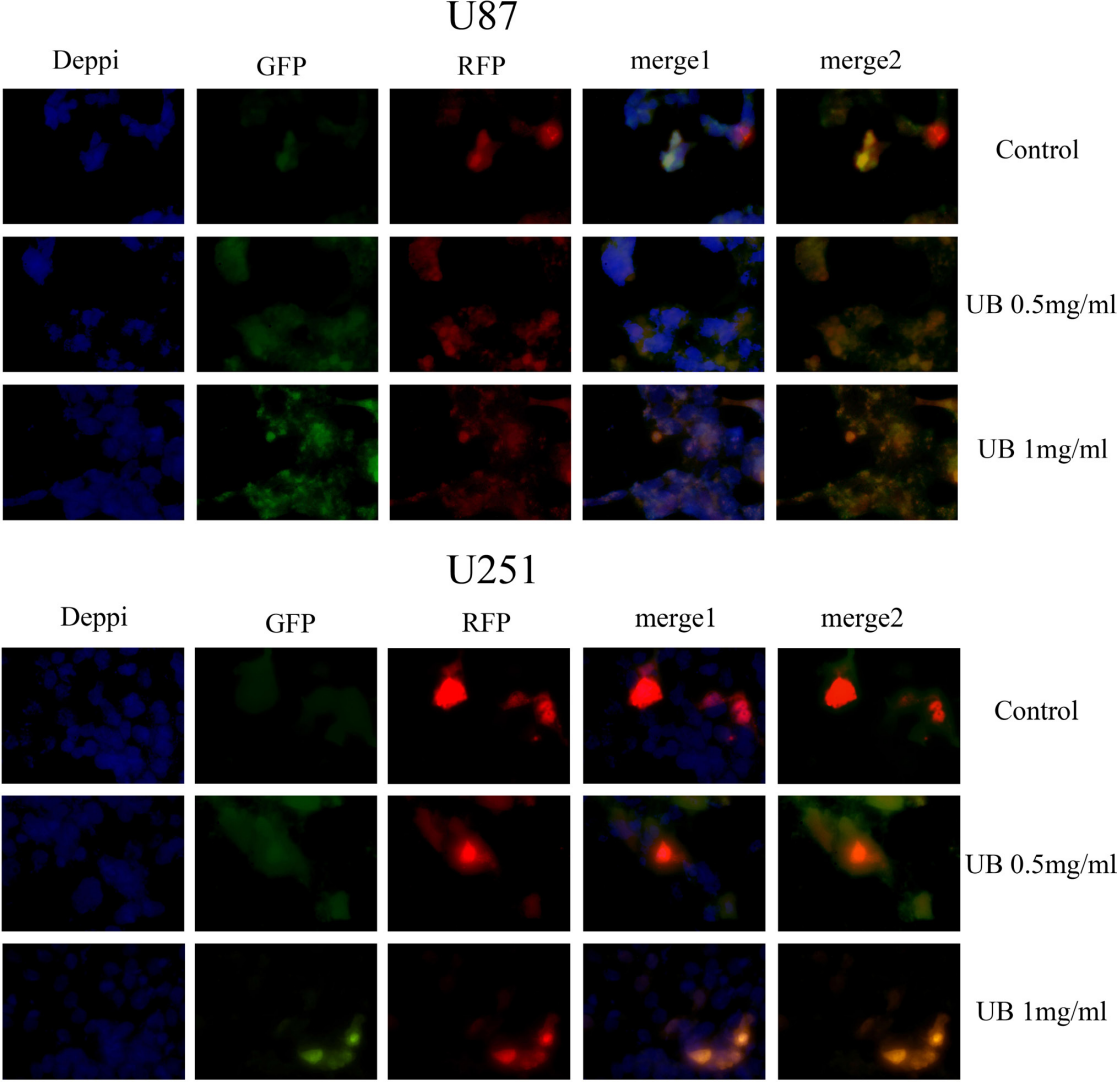
Ubenimex inhibited the autophagy flux in U87 and U251 cells Classic RFP-GFP-LC3 plasmid transfection was conducted to study autophgic flux level. RFP-GFP-LC3B was used to monitor various stages of autophagy. Image-based analysis was performed for autophagy. RFP-GFP sensor capitalizes on the pH difference between the acidic autolysosome and the neutral autophagosome and the pH sensitivity differences exhibited by GFP (green fluorescent protein) and RFP (red fluorescent protein) to monitor the progression from autophagosome to autolysosome, the change from autophagosome (neutral pH) to autolysosome (with an acidic pH) can be visualized by imaging the specific loss of GFP fluorescence, leaving only red fluorescence. U87 and U251 cells were treated with ubenimex at doses of 0.5 and 2 mg/mL for 16 h. The cells were harvested and re-cultured in 24-well plates. Then, mRFP-GFP-LC3 was added at the MOI of 50. Next, the cells were observed under a fluorescence microscope (Nikon Inc., Japan). Thus, the lysosomal pH was elevated compared to the normal autophagy flux (just red), indicating the inhibition of fusion of autophagosomes with lysosomes and preventing the subsequent lysosomal protein degradation [as shown in yellow (red+green)]. So, the autophagic flux was blocked by ubenimex. The experiment was performed in triplicate.

To further confirm the conclusion above, classic RFP-GFP-LC3 plasmid transfection was performed to study the autophgic flux level. Various stages of autophagy was monitored by using RFP-GFP-LC3B plasmid transfection (Figure 6). Image-based analysis was performed to determine autophagy, and RFP-GFP sensor capitalizes on the difference of pH between the autolysosome that are acidic and the neutral autophagosome. As pH sensitivity exhibited by green fluorescent protein (GFP) and (red fluorescent protein RFP), the progression from autophagosome to autolysosome, and from autophagosome (neutral pH) to autolysosome (with an acidic pH) can be visualized by imaging the specific loss of the GFP fluorescence, leaving only red fluorescence. Thus, if autophagic flux was blocked, lysosomal pH was elevated compared to the normal autophagic flux (just red), indicating the inhibition of the fusion of autophagosomes with lysosomes and preventing the subsequent lysosomal protein degradation [shown as yellow in the image (red+green)]. So, ubenimex in a dose-dependent manner inhibited autophagic flux in the glioma cells.

Besides, the proliferation was determined through WST-8 (Figure 1). Rapamycin, a well autophagic inducer, was added and the cell viability was apparently increased compared with ubenimex single treatment (*P* < 0.05). This suggested that ubenimex induced autophagy-related cell death in glioma cells.

### Besides autophagic inhibition, ubenimex induced apoptosis in glioma cells

**Figure 7:**
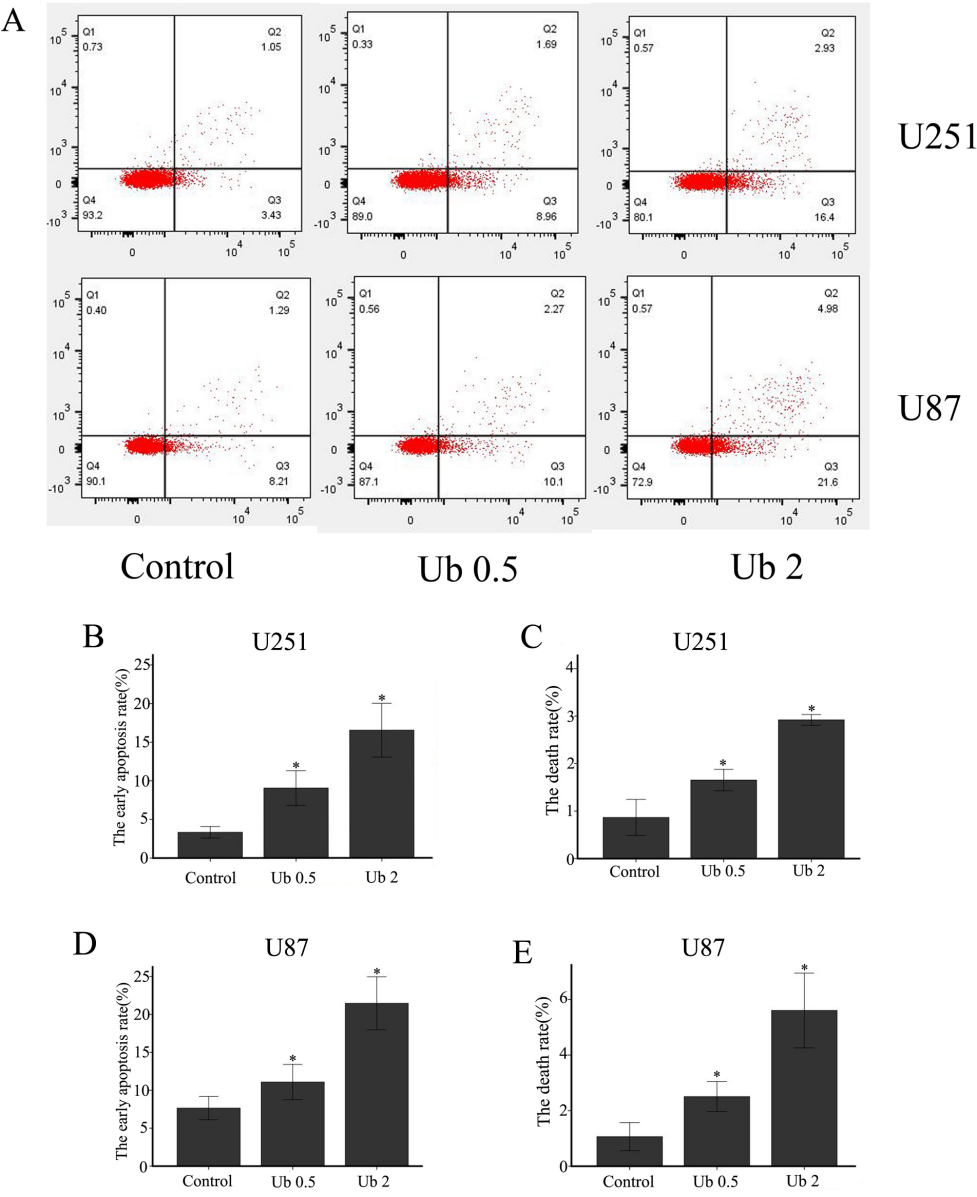
Ubenimex induced cell death and apoptosis in U87 and U251 cells (**A**) U87 and U251 cells were treated with ubenimex at doses of 0.5 and 2 mg/mL for 16 h. Annexin V-fluorescein isothiocyanate (FITC)/propidium iodide (PI) staining was used to examine cell death. (**B**–**E**) The death and apoptotic rates of U87 and U251 cells were increased with different doses of ubenimex. ^*^P < 0.05 versus the control. Data are expressed as mean ± standard deviation of three independent experiments.

Following treatment with different doses of ubenimex, western blot analyses were performed to examine the caspase-3 expression (Figure 4). After 16-h treatment with ubenimex, caspase-3 expression was motivated in a dose-dependent manner in the glioma cells. Besides, Annexin V-PE/7AAD staining was used to examine apoptosis and cell death (Figure 7). The present study demonstrated the level of apoptosis, which was increased following the treatment with ubenimex. Trypan blue double staining and WSK-8 results showed the level of cell death or cell viability (*P* < 0.05) (Figure 1), indicating that ubenimex could increase the apoptosis indirectly.

### Ubenimex promoted the proportion of cells in G2/M phase and demonstrated cell cycle arrest

**Figure 8:**
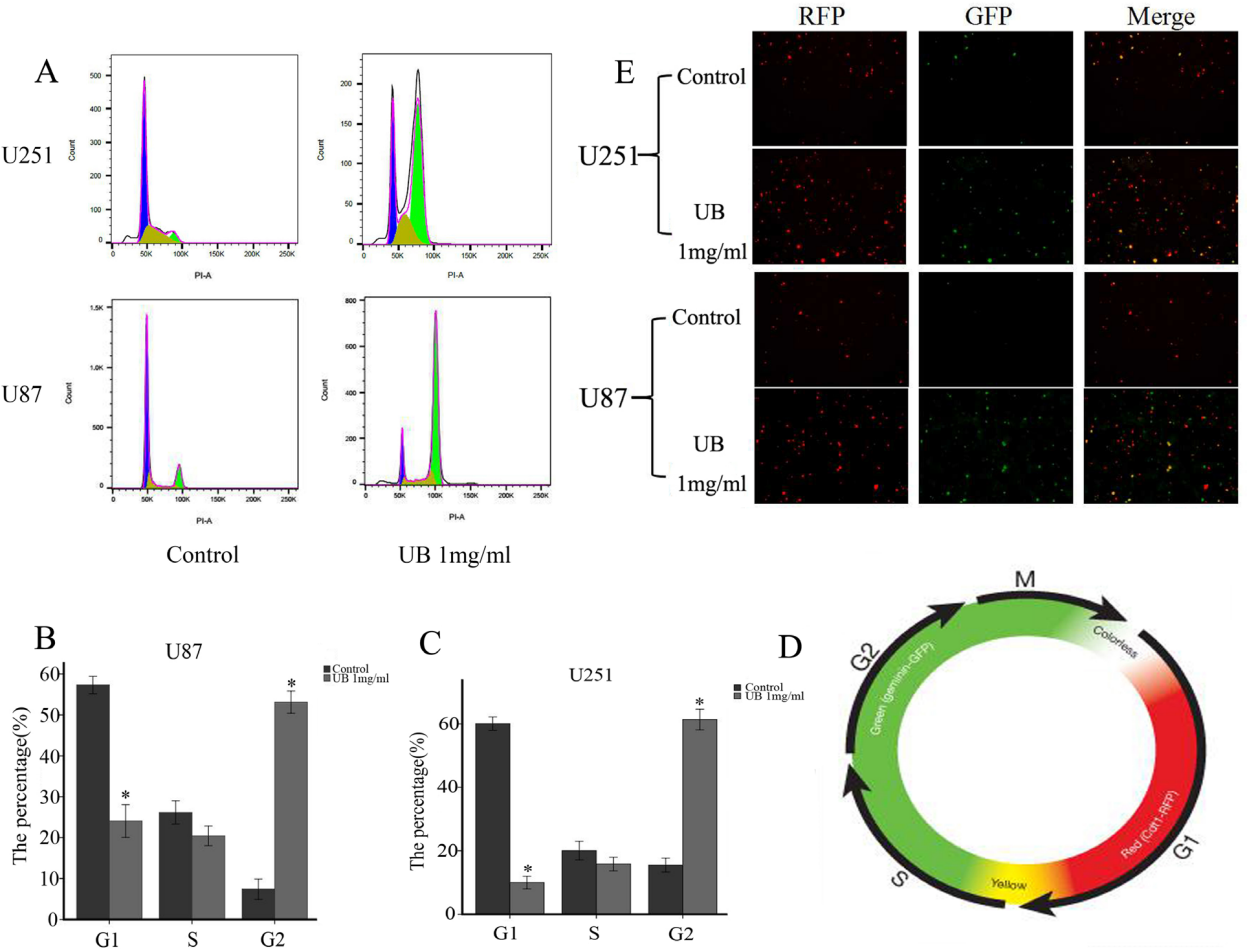
Ubenimex promoted the proportion of cells in G2/M phase The Premo FUCCI Cell Cycle Sensor consists of a fluorescent protein-based system that employs both red (RFP) and green (GFP) fluorescent protein fused to different regulators of the cell cycle: cdt1 and geminin. In the G1 phase of the cell cycle, geminin was degraded. Therefore, only cdt1 tagged with RFP was present and appeared as red fluorescence within the nuclei. In the S, G2, and M phases, cdt1 was degraded and only geminin tagged with GFP remained, resulting in cells with green fluorescent nuclei. During the G1/S transition, both proteins are present when cdt1 levels are decreased and geminin levels are increased and the nuclei appeared yellow fluorescent when the green and red images are overlaid. This dynamic color change, from red-through-yellow-to-green, represented the progression through cell cycle and division. (**A**) U87 and U251 cells were treated with ubenimex at doses of 0.5 and 2 mg/mL for 16 h. Stained cells were analyzed with flow cytometry on a FACSAria II. (**B**–**C**) The rates of U87 and U251 cells in different stages with different doses of ubenimex. (**D**) After treatment with ubenimex, the green significantly increased in U87 and U251 cells, showing a cell cycle arrest. (**E**) Color change in a cell cycle.

Mechanism of synergistic effects was further explored in combination with ubenimex by examining the distribution of cells in different stages of the cell cycle (Figure 8). Through PI staining, in both U87 and 251 cells, we found that the presence of ubenimex caused a significant increase in the proportion of cells in G2/M phase and a decrease in the G1 phase (Figure 8A).

We employed the Premo FUCCI Cell Cycle Sensor which consists a fluorescent protein-based system and has both RFP and GFP fused to different regulators of the cell cycle: cdt1 and geminin. Germinin is degraded during G1 phase of the cell cycle and only cdt1 was tagged with RFP and appeared in red fluorescence within the nuclei. However in the S, G2, and M phases, cdt1 was degraded and only geminin was tagged with GFP, resulting in cells with green fluorescent nuclei. During the G1/S transition, both proteins are present when the cdt1 levels decreases and geminin levels increases thus the nuclei appears yellow fluorescence when the green and red images superimpose (Figure 8D). This dynamic color change, from red-through-yellow-to-green, represented the progression through cell cycle and division. This result showed that ubenimex promoted the proportion of cells in G2/M phase, which showed the cell cycle arrest and glioma cell proliferation inhibition.

### Influences of autophagy and apoptosis might be related to the level of Akt pathways

Western blot was performed inorder to examine the expression of p-Akt in the glioma cells following treatment with ubenimex (Figure 4). It has been shown that Akt signaling pathways are involved in the regulation of autophagy [19, 20]. Therefore, we examined the phosphorylated proteins involved in the Akt signaling pathways and a similar trend as tumor cell death related protein was found. These suggested that ubenimex treatment inhibited the expression of Akt.

### Ubenimex inhibited the proliferation of glioma cells *in vivo*

**Figure 9:**
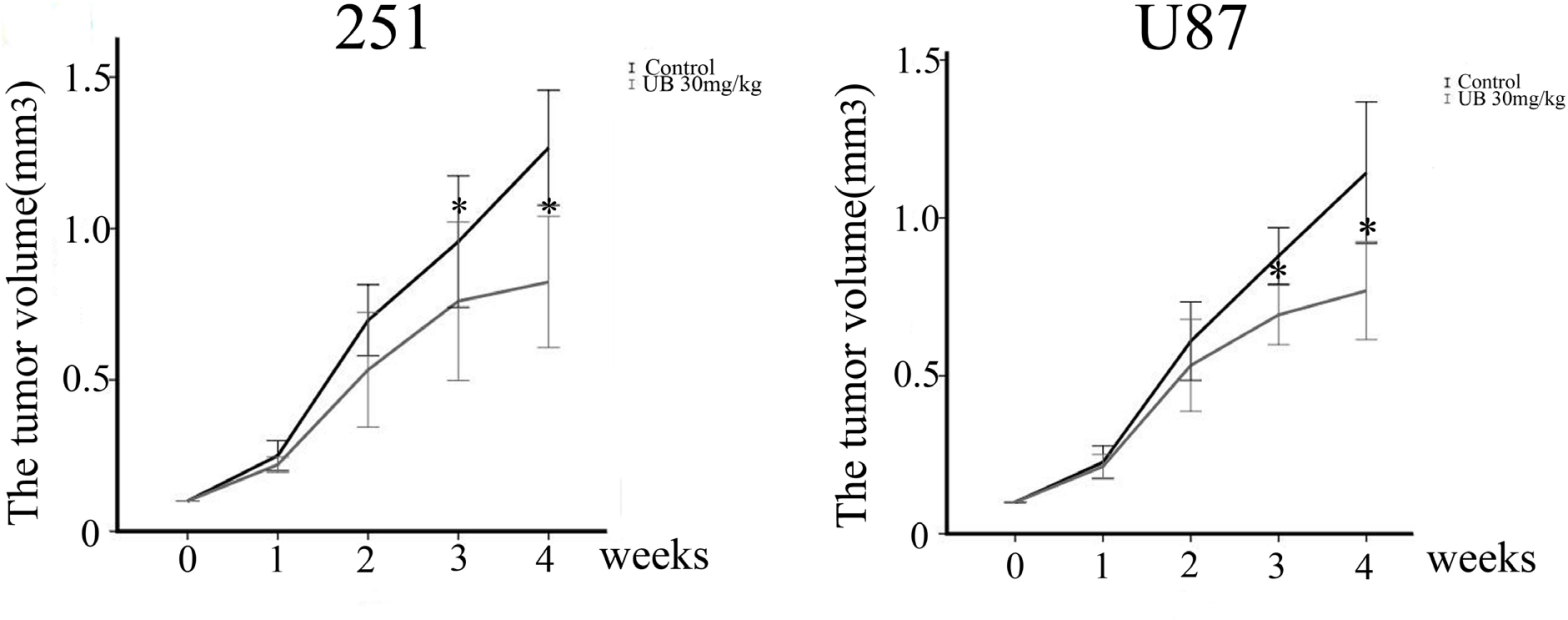
*In vivo* tumor growth assays using U87 and U251 tumor models Subcutaneous injection of 5 × 10^6^ U251 or U87 cells per nude mouse was carried out. Mice were treated with i) vehicle (corn oil) and ii) 30 mg/kg ubenimex. These treatments were administered 3 times/week for 4 weeks. Tumor lengths and widths were measured after each week. Tumor volume was derived as length × width 2/2. ^*^P < 0.05 vs. the control. Data are expressed as mean ± standard deviation of 3 independent experiments.

In order to evaluate whether ubenimex has the antitumor growth effect *in vivo*, tumors were induced by injecting U87 and U251 cells, respectively into nude mice. Body weight of the mice were charted weekly following treatment with ubenimex. None of the ubenimex treatment produced significant loss in the body weight, which constituted a sign of toxicity (data not show). The tumor volume in the nude mice was significantly reduced following ubenimex treatment (Figure 9, *P* < 0.05). Therefore, our study clearly demonstrated the potential of ubenimex in inhibition of U87 and U251 cell proliferation *in vivo*.

## DISCUSSION

Our previous study has demonstrated the function of ubenimex in enhancing JQ1 treatment sensitivity of glioma cells by blocking autophagic degradation of HEXIM1 [9]; however, the detailed mechanism of autophgy regulation by ubenimex remains unclear. Johannes Klose's study suggested that this might be via attenuation of the late stages of HCC autophagy-autophgic flux which might have led to the impairment in recycling and therefore accumulation of dysfunctional mitochondria, and was associated with the induction of apoptosis [10]. Our results demonstrated that ubenimex inhibited autophagic flux, leading to autophagic substrate (LC3-II) accumulation, as well as autophagosome increase as monitored by EM. This in turn led to apoptosis and cell growth inhibition. However, it was unclear whether this was due to enhanced autophagosome generation from increased autophagic activity or from suppressed autophagosome maturation [21]. To address this question and to assess the autophagic flux by western blot, we estimated the level of autophagolysosomal degradation of P62. Results revealed that P62 degradation was blocked by ubenimex, indicating its role in blocking autophagic flux. Another approach was also employed to verify this theory, classic RFP-GFP-LC3 plasmid transfection was performed to study the autophagic flux level. Data showed that ubenimex significantly blocked the glioma cell's autophagic flux.

The crosstalk between autophagic flux and cell cycle remains unclear in glioma cells. A significant G2/M arrest by ubenimex treatment, and also found that the cells was affected with autophagic flux inhibition. Shift between various cell cycles is a process that involves-cellular remodeling and requires high energy demand, especially in cancer cells. While autophagic degradation is essential in cleaning and recycling damaged organelles as well as providing energy. Autophagic flux may play a pivotal role in the whole autophagy progression. Tasdemir *et al.* have shown that various autophagy inhibitors/inducers can lead to differential effects in a cell cycle-dependent way [22]. Thus, it is important to study the role of autophagic flux inhibition in G2/M cell cycle arrest of glioma cells by ubenimex. In our study, ubenimex arrested glioma cells in G2/M cell cycle and autophagic flux was inhibited. This was consistent with the cell growth and autophagy coordination of each other [23] and the autophagy regulation mechanism was cell cycle dependent [22]. It has been shown that combination therapy of autophagy inhibitors and chemodrugs that block cells in mitosis can yield better anti-tumor efficacy [24, 25]. Ubenimex can both work as autophagic flux inhibitor as well as arrest the G2/M phase, showing a great potential in the treatment of glioma.

Inhibition of autophagic flux promoted cell apoptosis [26]. Recent studies suggested that autophagy and apoptosis are connected. Primary function of autophagy is to recycle the damaged proteins, organelles and to provide energy to nourish depleted cellular components [27]. While impaired autophagy leads to decreased renewal of cell organelles and energy providing, contributing to cell apoptosis. Our study was consistent with this theory and verified that blocking of autophagic flux by ubenimex induced apoptosis in glioma cells. Apoptosis is a process that involves caspase-dependent cell death, whereas autophagy is associated involves caspase independent cell death [28], and both could cause tumor cell death. However, autophagy associated cell death is different from apoptosis [29]. In our previous publication, we demonstrated that ubenimex inhibited autophagy leading to cell death in glioma cells [30]. Here, we found the mechanism of these through autophagic flux. We found that as the dose of ubenimex increased, the autophagic flux was inhibited. In the present study, apoptosis of glioma cells was measured through Annexin V–PE/7AAD staining and Western blot. Results demonstrated increased apoptotic rate of U87 and U251 cells with ubenimex treatment. Results of Annexin V-PE/7AAD and trypan blue staining showed that the number of dead cells was increased in a ubenimex dose-dependent manner. These effects were attenuated when rapamycin was subjected to stimulate autophagy, indicating that autophagy inhibition induced cell death in glioma cells. Therefore, this study concluded that ubenimex could cause cell death based on autophagic flux inhibition and apoptosis in glioma cells.

APN, also known as CD13, has been found to be overexpressed in many malignant tumors, such as lung and breast cancers [31, 32], and also played an important role in tumor cell proliferation [33]. Ubenimex has been demonstrated to induce cell death in RCC and prostate cancer cells however, it is plausible that this phenomenon might be due to the attenuation of APN activity and upregulation of autophagic cell death [16, 17]. However, Mawrin *et al.* [34] indicated lower expression of APN in brain tumor. Probably, this might be the reason that prompted limitation on research reports on the role of APN in brain tumor. However, Sina *et al.* [35] showed correlation of APN expression with tumor-associated angiogenesis in the brain, which was closely related to tumor growth. Thus, APN expression was measured using Western blot analysis after treatment with different doses of ubenimex in U251 and U87 cells. Significant APN inhibition was found after the treatment, indicating that ubenimex could inhibit APN expression in glioma cells. Moreover, the trend was closely related to apoptosis and death in U87 and U251 cells. Therefore, APN might be a contributing factor in inducing apoptosis in U87 and U251 cells using low doses of ubenimex.

APN also participated in tumor cell cycle. The highest APN-expression rate had been shown to be in the G0/G1 phase [36], indicating inhibition of APN abrogated dormancy of APN-positive cell via pushing them forward from G0 to enter the cell cycle. DNA damage occurs in cells undergoing apoptosis however, prosurvival mechanism by activation of G2 checkpoint gives time to the cells to repair their DNA. If not then the impeding cell cycle arrest in DNA-damaged cells normally leads to cell death [37]. The failure happened via mitosis entry even in the presence of DNA damage or checkpoint over activation [19, 38]. Not all cancer cells die of apoptosis. For example, glioblastoma multiforme (GBM), are intrinsically resistant to apoptosis and more sensitive to other mechanisms of cell death, such as autophagy [39, 40]. According to Murakami H *et al.* study, ubenimex alone showed no effect on-the cell cycle in HCC [41]. We tested the function of ubenimex in regulating the tumor cell cycle in glioma cells. Results revealed that ubenimex caused a dramatic increase in the proportion of cells in G2/M phase, suggesting that ubenimex could inhibit the proliferation of glioma cells.

Besides, our previous paper has shown that ubenimex could inhibit cell migration/ invasion in metastatic prostate cancer cells by downregulating APN [17]. APN expression is reported to be related to various cellular processes, such as motility, attachment, invasion/metastasis of various cancers [41]. Besides, high level of APN expression is regarded as an prognostic risk for metastatic tumor development in lung, pancreas and colon cancers [20, 41]. Thus, we tested glioma cells APN expression by using western blot analysis, after treatment with varying doses of ubenimex and found that the expression was significantly inhibited by the increased dose of ubenimex. Also, it was closely associated with the migration/invasion of glioma cells. Therefore, we concluded that APN inhibition by ubenimex could inhibit cell metastases in glioma cells.

(PI3K)/Akt signaling pathway is primarily involved in two major ways in this study. One being phosphoinositide 3-kinase (PI3K)/Akt which although primarily involved in cell death, it is considered to deliver survival signals to protect cells from apoptosis [14]. However, other hand Akt signaling pathway plays a key role in connecting apoptosis with autophagy processes [42]. Reduction of PI3K/Akt signaling was associated with the level of autophagy and apoptosis [43–44], which was closely related to cell death. Additionally, it has also been reported that stress is a major component that activates the Akt signal transduction pathway in tumor cells, resulting in protective autophagy [45]. Also, Lou *et al.* [46] reported that quercetin nanoparticles could influence autophagy and apoptosis in human neuroglioma cells through LC3/caspase-3 activation and Akt/mTOR signaling suppression. In this study, ubenimex was found to influence Akt expression, and these worked as regulators of autophagy in U251 and U87 cells. The Akt pathways also affected the level of apoptosis when treated with high dose of ubenimex.

In summary, ubenimex induced glioma cell death via both autophagic flux inhibition and induction of apoptosis. G2/M phase arrest was also involved in ubenimex induced proliferation inhibition, indicating that there might be a cross-talk between autophagy regulation and cell cycle arrest. As an APN inhibitor, ubenimex also shows effect in inhibiting glioma cells’ migration and invasion. Akt pathway was verified to be involved in these effects caused by ubenimex treatment alone. Therefore, this study concluded that ubenimex might have potential to be one of the primary candidate in adjunctive therapy in the treatment of glioma.

## MATERIALS AND METHODS

### Tumor cell lines

Glioma cell lines U251 and U87 were purchased from the Chinese Academy of Sciences Cell Bank. Cells were maintained in Dulbecco's modified Eagle medium (DMEM), a high-glucose medium (Macgene, China) supplemented with 1% penicillin–streptomycin and 10% fetal bovine serum (Shanghai ExCell Biology). The cells were incubated at 37°C in a humidified atmosphere with 5% CO_2_.

### WST-8 cell proliferation assay

U251 and U87 cells in an exponential phase of growth were harvested and seeded into 96-well plates at a density of 5000 cells/well in the DMEM high-glucose medium supplemented with different concentrations of ubenimex. After 24 h of culture, 10 μL of WST-8 solution (WST-8 cell proliferation and cytotoxicity assay kit; Dojindo, Japan) was added into each well. The plates were then incubated for an additional 3 h at 37°C, and the absorbance was determined using a microplate reader (EL340; Bio-Tek Instruments, MA, USA) at 450 nm.

### Trypan blue staining

The cells were cultured in six-well plates for 24 h and then treated with different doses of ubenimex for 16 h. After the indicated treatment times, cell suspension and 0.4% trypan blue solution were mixed in 9:1 ratio. After three minutes, the counting plate containing the live cells and dead cells were counted. Cell viability was measured using the following formula: living cell rate (%) = living cell total/(total number of living cells + dead cells) ×100%.

### Western blot analysis

To determine the expression levels of LC3B, and phosphor-Akt-ser473 (p-Akt-ser473), the samples were suspended in radioimmunoprecipitation assay (RIPA) buffer supplemented with phenylmethylsulfonyl fluoride and phosphatase inhibitor (100:1:1) for protein extraction. The samples were then centrifuged at 12,000 rpm at 4°C for 30 min, and the supernatants were recovered for analysis. The protein concentrations were determined using the Bradford protein method and the bicinchoninic acid protein assay kit (Sigma, MO, USA). Protein (40 μg) was electrophoresed on a precast bis-Tris polyacrylamide gel (12% or 10%), and transferred onto a polyvinylidene difluoride membrane. Membranes were blotted with rabbit anti-APN (1:1000), rabbit anti-LC3B (1:500), rabbit anti-P62, rabbit anti-beclin-1 (both from Abcam, USA), rabbit anti-p-Akt, rabbit anti-Akt (CST, USA), and mouse anti-actin (1:5000; BL005A; Biosharp, Beijing, China), followed by horseradish peroxidase-conjugated secondary antibodies (1:5000; ZsBio, Beijing, China). Immunoblots were visualized using enhanced chemiluminescence (LAS-4000).

### Electron microscopy

Glioma cells were treated with 2 mg/mL ubenimex for 16 h and then fixed with 3% glutaraldehyde and 2% paraformaldehyde in 0.1 M phosphate-buffered saline buffer for 30 min, post-fixed with 1% osmium tetroxide for 1.5 h, and washed and stained with 3% aqueous uranyl acetate for 1 h. Next, the cells were dehydrated in an ascending series of ethanol and acetone and then embedded in araldite. Ultrathin sections were cut using a Reichert ultramicrotome, double-stained with 0.3% lead citrate, and examined under a JEOL 1200EX electron microscope (Japan).

### Cell cycle analysis with propidium iodide staining

For cell cycle analysis, each type of cells were incubated with different doses of ubenimex for 16 h, and then fixed in 70% ethanol on ice. After centrifugation, cells were stained with 50 mg/ml propidium iodide (PI) solution (Dojindo Molecular Technologies, kumamoto, Japan) and 0.1 mg/ml RNase A (Invitrogen). Stained cells were analyzed using flow cytometry on a FACSAria II. Histogram was constructed with data from at least 20,000 events. Besides, in the G1 phase of the cell cycle, geminin was degraded. Therefore, only cdt1 tagged with RFP was present and appeared as red fluorescence within the nuclei. In the S, G2, and M phases, cdt1 was degraded and only geminin tagged with GFP remained, resulting in cells with green fluorescent nuclei. Flow cytometric analyses were performed using FlowJo software.

### Matrigel migration assay

Migration assays were performed using Transwell chambers. Control untreated cells or cells treated with ubenimex (0.5, or 1 mg/ml for 16 h) were trypsinized. Cells were plated in the upper wells in serum-free medium, while medium with 10% FBS was added to the lower well as a stimulus. After 36 h of incubation, the cells on the Matrigel side of the chambers were removed using a cotton swab. The inserts were fixed in methanol and stained using hematoxylin and eosin (H&E) staining. The number of invading cells attached to the other side of the inserts was quantified under a light microscope using 8 random fields at a magnification of ×200. The experiment was performed in triplicate. Matrigel invasion assay

Invasion assays were performed using Transwell chambers that were pre-coated with 40 μl of 1 mg/ml Matrigel matrix (BD Biosciences, Bedford, MA, USA). Control untreated cells or cells treated with ubenimex (0.5 or 1 mg/ml for 16 h) were trypsinized, and then the cells were plated in the upper wells in serum-free medium, while medium with 10% FBS was added to the lower well as a stimulus. After 36 h of incubation, the cells on the Matrigel side of the chambers were removed using a cotton swab. The inserts were fixed in methanol and stained using H&E staining. The number of invading cells attached to the other side of the inserts was quantified under a light microscope using 8 random fields at a magnification of ×200. The experiment was performed in triplicate.

### Detection of autophagic flux

mRFP-GFP-LC3 was purchased from Hanbio Biotechnology Co., Ltd. Cells were cultured in six-well plates for 24 h and then treated with different doses of ubenimex for 16 h. The cells were harvested and re-cultured in 24-well plates. Then, mRFP-GFP-LC3 was added at the MOI of 50. Next, the cells were observed under a fluorescence microscope (Nikon Inc., Japan). The experiment was performed in triplicate.

### Annexin V-fluorescein isothiocyanate (FITC)/propidium iodide (PI) staining

Apoptotic cells were quantified (%) using annexin V-FITC/PI kit (Nanjing KeyGen Biotech, Co.Ltd., Nanjing, China) according to the manufacturer's instructions and detected by flow cytometry. The cells were harvested after 16 h of treatment with different doses of ubenimex. Next, the cells were resuspended in the binding buffer and incubated with annexin V-FITC/PI in the dark for 15 min. A total of 5,000 cells/sample were analyzed using FACSCalibur or EPICS XL flow cytometer (BD Biosciences, Franklin Lakes, NJ, USA).

### Statistical analysis

The data were statistically analyzed using Student's *t*-test, Chi-square test, or Fisher's exact test using the SPSS software version 19.0 (IBM SPSS, NY, USA). A *P* value < 0.05 was considered to be statistically significant.
